# Is it time to abandon the biological species concept? No

**DOI:** 10.1093/nsr/nwaa109

**Published:** 2020-06-02

**Authors:** Roger K Butlin, Sean Stankowski

**Affiliations:** Department of Animal and Plant Sciences, University of Sheffield, UK; Department of Marine Sciences, University of Gothenburg, Sweden; Department of Animal and Plant Sciences, University of Sheffield, UK

The *Oxford English Dictionary* defines a concept as, ‘an idea of a class of objects, a general notion’. It follows from this definition that a concept cannot be rejected in the way that a hypothesis might be rejected if its predictions are inconsistent with observations. Instead, a concept must be judged by its heuristic value: does it help in making sense of the natural world?

The biological species concept (BSC) was designed to aid understanding of biological diversity, particularly the ubiquitous observation that sexually reproducing organisms exist in more or less distinct phenotypic and genetic clusters rather than in a continuum of forms. It does so by focusing attention on the contrast between successful interbreeding within groups and reproductive isolation between them. Distinct groups can form in other ways, and can occur in organisms that lack regular sexual reproduction. This has led to alternative conceptualizations of the units of diversity [1,2]. However, the fact that the BSC is highlighted in every biology textbook and lecture course, more than 80 years after it was introduced and formalized [3,4], is testament to its continued utility. Perhaps most importantly, the BSC identifies a research programme for understanding the origin of biological diversity by equating the process of speciation with the evolution of reproductive isolation. This has been the foundation of a huge body of research in evolutionary biology since the neo-Darwinian synthesis, which has led to a much deeper understanding of species and speciation, although the job is certainly not yet completed.

Understanding the process by which species form can certainly aid in understanding the nature of species. Indeed, concepts in general aim to be ‘fundamental links bridging observable patterns and inferred processes’ [1] and this is certainly true of the BSC. However, Wang *et al*. [5] seek to go a step further: rather than linking the existence of discontinuities among species with the processes of gene flow, natural selection and the evolution of reproductive isolation, which is a standard interpretation of the BSC, they make the BSC dependent upon a particular mode of speciation, namely ‘allopatric speciation’. Mayr [6] did not make this connection. Indeed, he discussed species concepts (Chapter 2) and speciation processes (Chapters 15–17) in separate parts of his book. Nor has the connection been made in more recent monographs [7,8]. The link made by Wang *et al*. [5] is problematic in principle. Suppose it can be shown that two populations are now reproductively isolated and have acquired that isolation without any period of spatial separation, would Wang and co-workers conclude that the populations belong to the same species? This is, actually, not just a theoretical problem: the origin of polyploid species provides multiple concrete examples (e.g. [9]). Linking the BSC to a particular ‘mode of speciation’ is also problematic because of the difficulty of defining and distinguishing these modes [10]. In reality, speciation is complex, extended over time and space and involving multiple processes, leading to a wide range of possible routes towards complete reproductive isolation [11,12]. The accumulated evidence [7,8,12,13] suggests that many of these paths have actually been followed.

Wang *et al*. [5] suggest that an alternative to the BSC is a ‘genic view’ of species where ‘species are defined by a set of loci that govern the morphological, reproductive, behavioral and ecological characters’. As it stands, this definition is incomplete because it does not specify what sets the significant characters apart from the rest of the phenotype or what features of the set of loci distinguish species. However, it is clear from their further discussion that the characters in question are those that contribute to reproductive isolation (‘fitness-reducing upon introgression’) and that these loci should define distinct genetic clusters despite potential for gene flow. If the BSC allows for incomplete reproductive isolation, as is commonly accepted (e.g. [1,7,14]), then there is actually no difference between this genic view and the BSC, unless the BSC is tied to allopatric accumulation of reproductive isolation and the genic view is not. To answer the question posed by Wang *et al*. [5]: No, it is not time to abandon the BSC.

Nearly 20 years ago, Wu [15] proposed a ‘genic view of *the process of speciation*’. This proposal struck a chord and fig. 1 from Wu's paper has been very widely reproduced. It describes snapshots in the evolution of reproductive isolation from the appearance of the first barriers to gene flow to the complete absence of successful interbreeding (described as ‘Stages’, perhaps with the unhelpful implication of discontinuities in a continuous process). The underlying idea of an initially semi-permeable barrier that can evolve to exclude a larger and larger proportion of the genome has great heuristic value. It was already present in the hybrid zone literature [16] and is now widely used (e.g. [17]). Wang *et al*. [18] provide a nice example from mangroves in their companion paper. However, this idea does not challenge the BSC. What it does do is to require careful consideration of the meaning of reproductive isolation, which is, of course, central to understanding and applying the BSC. When reproductive isolation is complete, there is a complete barrier to gene flow throughout the genome. When there is no barrier at any locus, there is no reproductive isolation. For partial reproductive isolation, there is no clear relationship. Two taxa may have a probability of mating of 0.2 and F1 hybrid fitness of 0.3, relative to parental fitness, leading to an estimate of reproductive isolation of 1–0.2 * 0.3 = 0.94 [19]. However, this tells us very little about the barrier to gene flow at any particular locus in the genome: it may be very low for a large proportion of loci that are unlinked to barrier loci and high at a few major-effect loci, or it may be relatively uniform across the genome if the barrier traits are highly polygenic. The reverse is also true: measuring the barrier to gene flow at one locus by population genetic approaches does not provide an estimate of reproductive isolation. Understanding these relationships is greatly aided by a genic view of speciation but they do not require a new species concept.
